# Towards the Development of the Clinical Decision Support System for the Identification of Respiration Diseases via Lung Sound Classification Using 1D-CNN

**DOI:** 10.3390/s24216887

**Published:** 2024-10-27

**Authors:** Syed Waqad Ali, Muhammad Munaf Rashid, Muhammad Uzair Yousuf, Sarmad Shams, Muhammad Asif, Muhammad Rehan, Ikram Din Ujjan

**Affiliations:** 1 Data Acquisition, Processing & Predictive Analytics Lab, National Center in Big Data and Cloud Computing (NCBC), Ziauddin University, Karachi 74600, Pakistan; munaf.rashid@zu.edu.pk; 2 Department of Biomedical Engineering, Sir Syed University of Engineering and Technology, Karachi 75300, Pakistan; 3 Department of Mechanical Engineering, NED University of Engineering and Technology, Karachi 75270, Pakistan; uzairned@hotmail.com; 4 Institute of Biomedical Engineering & Technology, Liaquat University of Medical and Health Sciences, Jamshoro 76060, Pakistan; ikramujjan@lumhs.edu.pk; 5 Faculty of Computing and Applied Sciences, Sir Syed University of Engineering and Technology, Karachi 75300, Pakistan; 6 Department of Electronic Engineering, Sir Syed University of Engineering and Technology, Karachi 75300, Pakistan; murehan@ssuet.edu.pk

**Keywords:** CDSS, respiratory disease, CNN, lung sound analysis, crackle and wheeze

## Abstract

Respiratory disorders are commonly regarded as complex disorders to diagnose due to their multi-factorial nature, encompassing the interplay between hereditary variables, comorbidities, environmental exposures, and therapies, among other contributing factors. This study presents a Clinical Decision Support System (CDSS) for the early detection of respiratory disorders using a one-dimensional convolutional neural network (1D-CNN) model. The ICBHI 2017 Breathing Sound Database, which contains samples of different breathing sounds, was used in this research. During pre-processing, audio clips were resampled to a uniform rate, and breathing cycles were segmented into individual instances of the lung sound. A One-Dimensional Convolutional Neural Network (1D-CNN) consisting of convolutional layers, max pooling layers, dropout layers, and fully connected layers, was designed to classify the processed clips into four categories: normal, crackles, wheezes, and combined crackles and wheezes. To address class imbalance, the Synthetic Minority Over-sampling Technique (SMOTE) was applied to the training data. Hyperparameters were optimized using grid search with k−fold cross-validation. The model achieved an overall accuracy of 0.95, outperforming state-of-the-art methods. Particularly, the normal and crackles categories attained the highest F1-scores of 0.97 and 0.95, respectively. The model’s robustness was further validated through 5−fold and 10−fold cross-validation experiments. This research highlighted an essential aspect of diagnosing lung sounds through artificial intelligence and utilized the 1D-CNN to classify lung sounds accurately. The proposed advancement of technology shall enable medical care practitioners to diagnose lung disorders in an improved manner, leading to better patient care.

## 1. Introduction

Respiratory disorders are commonly regarded as complex disorders to diagnose because of their multi-factorial nature, encompassing the interplay between hereditary variables, comorbidities, environmental exposures, and therapies, among other contributing factors [1]. Numerous respiratory ailments, including chronic obstructive pulmonary disease (COPD), asthma, cystic fibrosis (CF), and sleep apnea, as well as malignant disorders such as lung cancer and respiratory infections such as influenza or COVID-19, have a significant impact on persons across all age cohorts [2,3]. In addition to providing acute respiratory treatment, these disorders, as mentioned earlier, provide a formidable barrier to allocating health system resources, necessitating meticulous and effective management.

One way to analyze respiratory disorders is through the auditory evaluation of abnormal lung sounds through a non-invasive diagnostic approach using a stethoscope or microphone [4]. The sounds produced by the pulmonary system have the potential to provide valuable information on the presence and severity of respiratory disorders. However, examining lung sounds is a specialised and subjective task that requires considerable expertise and experience to analyze the acoustic signal effectively. Asthma and chronic obstructive lung illnesses often cause wheezing, as this symptom indicates persistent aberrant respiratory sounds. Meanwhile, medical professionals clinically correlate pneumonia, bronchitis, and other medical problems with intermittent adventitious crackling sounds. In recent decades, extensive research efforts have been dedicated to investigate the detection and classification of these entities. The categorization of wheeze, crackle, and normal sounds necessitates significant refinement due to the presence of artifacts and the constraints of feature extraction techniques [5]. Trainees, such as interns and residents, may encounter challenges in accurately identifying respiratory sounds. Therefore, digital respiratory sounds provide very useful data for disease detection, making them particularly relevant for intelligent diagnostics.

In recent years, significant efforts have been made in the research on the automated classification of lung sounds. Mazić et al. [6] utilized a feature vector composed of Mel-frequency cepstral coefficients (MFCC), entropy, and kurtosis features. These were incorporated into a Support Vector Machine (SVM) framework with two parallel SVMs, which were each assigned gamma values of 2.0 and 5.0. The fusion of predictions from these SVMs was used to distinguish between respiratory sounds with wheezes and those without. Conversely, Matsutake et al. [7] applied hidden Markov models (HMMs) and maximum likelihood estimation techniques to classify respiratory sounds as either normal or pathological. Sen et al. [8] compared the performance of a Gaussian mixture model and SVM in distinguishing normal from abnormal lung sounds. Recently, there has been considerable research in developing deep learning-based models and algorithms for detecting and classifying lung sounds [9,10]. These advancements hold significant promise in the medical field, as deep learning algorithms can capture complex data patterns, enhancing the accuracy when diagnosing respiratory conditions in noisy healthcare environments [10].

One of the pioneering efforts in this field was conducted by Acharya and Basu [11], who introduced a deep Convolutional Neural Network–Recurrent Neural Network (CNN-RNN) architecture for classifying respiratory sounds using Mel spectrograms. Following this, Amose et al. illustrated the use of deep learning methods for classifying the lung sounds [11,12]. Expanding on this foundation, [12,13] explored the implementation of both machine learning and deep learning strategies to categorize different lung sound types, including crackles, wheezes, and normal breath sounds. Furthermore, another study [11] emphasized the utilization of deep learning models for lung sound classification using Mel spectrograms.

Demir et al. [14] integrated the deep features obtained through a CNN model using the linear discriminant analysis–random subspace ensembles (LDA-RSE) to distinguish various lung sounds. This approached significantly improved the diagnostic precision. T. Nguyen et al. [15] utilized the pre-trained ResNet model as the main framework to differentiate between lung sounds and respiratory diseases. They highlighted the promising role of deep learning in healthcare, especially in enhancing the diagnostic and treatment methods. Pham et al. [16] utilized the improved CNN architecture by integrating a mix of experts blocks. This approach enhanced the model’s ability to identify the complex patterns in lung sounds. They employed a variety of features that include Mel spectrograms, gamma-tone-based spectrograms, and rectangular constant Q transform features, which led to exceptional accuracy in classifying respiratory sounds.

### Current Research Status of 1D-CNN in Lung Sound Classification

1D-CNN has become increasingly important in sound classification. These networks are uniquely suited to process sequential data, such as time series or signal data, making them particularly useful for analyzing respiratory sounds [17,18,19].

A significant benefit of using 1D-CNNs for classifying lung sounds is their capacity to automatically extract features from raw audio signals, reducing the time needed for feature engineering. The 1D convolution operation allows for the detection of patterns and time domain features, which is essential for distinguishing various lung sound types [20]. Additionally, 1D-CNNs offer computational efficiency advantages, making them suitable for real-time applications and deployment on resource-constrained devices. This efficiency is especially important in clinical environments, where rapid analysis and decision-making are essential.

Recent studies have shown the benefits and potential applications of 1D-CNNs in the classification of sounds. For instance, Kim et al. [21] utilized a 1D-CNN model to classify snoring sounds, achieving high accuracy and efficient feature extraction. By using the multi-feature extraction techniques within a 1D-CNN framework, they successfully captured the temporal characteristics of snoring sounds. Furthermore, 1D-CNNs excel at analyzing acoustic or time-domain features, such as frequency spectrum, intensity, and duration. This makes them particularly valuable for the automated evaluation of lung sounds and the development of clinical decision support systems (CDSS) [22].

Petmezas et al. [9] developed a hybrid CNN-LSTM network that incorporated a focal loss function for the automated classification of lung sounds. This approach combined the 1D-CNN’s ability to identify spatial patterns with the LSTM network’s capacity to model long-term dependencies. The focal loss function was specifically designed to address class imbalance issues that are common in lung sound datasets.

Recent studies have demonstrated the promising applications of 1D-CNNs in lung sound classification across different domains [23,24,25,26]. Researchers have utilized 1D-CNNs to effectively classify respiratory sounds such as wheezes, crackles, and normal breathing, contributing to the diagnosis of respiratory diseases like asthma and COPD [9,19,22,23,24,25,26].

In summary, the field of lung sound classification using deep learning has made significant strides in recent years. Researchers have explored diverse architectures, techniques, and feature representations to improve diagnostic accuracy and efficiency.

From the state-of-the-art CNN-RNN hybrid to the modern mixture of experts, the breadth of methods used indicates the multifaceted nature of this research domain. Moving forward, this research aims to develop an automated system for classifying respiratory sounds, focusing on wheezing and crackling, using 1D-CNN to help diagnose and monitor respiratory diseases.

The main novelties of this work are detailed below:

- The data set was cleaned, pre-processed and segmented into wheezes and crackles.
- For the first time, the classification of wheeze and crackle signals was achieved using 1D-CNN.
- The class imbalance issue in the lung sound was overcome using the Synthetic Minority Over-sampling Technique (SMOTE).
- The analysis was performed on 1D-CNN, which significantly reduced the computational requirements, which allowed for its implementation in smaller hardware.

The rest of the paper is organised as follows: Section 2 discusses the materials and methods used in this study, including the ICBHI 2017 breathing sound database, the preprocessing steps applied to the dataset, and the proposed 1D-CNN architecture for lung sound classification. This section also covers the techniques used to address class imbalance, hyperparameter tuning using grid search with k−fold cross-validation, and the overall research methodology. Section 3 presents the results of the study, including the classification performance of the 1D-CNN model on four different categories of lung sounds (normal, crackles, wheezing, and combined crackles and wheezing). It also provides a comparison of the proposed model with state-of-the-art research in the field and discusses the results of k−fold cross-validation experiments. This section discusses the significance and implications of the findings. Finally, Section 4 summarizes the main contributions of the research, highlights the strengths and limitations of the proposed approach, and calls for further investigation and improvement of the developed Clinical Decision Support System (CDSS) for the diagnosis of respiratory disorders.

## 2. Materials and Methods

### 2.1. Database

In this research paper, we used the ICBHI 2017 Breathing Sound Database as a source for the respiratory sounds analysis. This widely recognized and publicly accessible lung sound dataset serves as a benchmark for researchers in the field [27]. The database is frequently cited in the literature and comprises a total of 5.5 h of recordings, further categorised into 920 audio samples originating from 128 individuals, as shown in Table 1. The annotation process for these audio samples was conducted manually by medical professionals. The cumulative number of respiratory cycles amounts to 6898, comprising 3642 instances of normal breathing, 1864 instances of crackles, 886 cases of wheezes, and 506 instances of combined crackles and wheezes. In this study, we include normal, crackles, wheezes, and combined crackles and wheezes as lung sound categories to provide a comprehensive evaluation of the proposed 1D-CNN model.

### 2.2. Pre-Processing

**Figure 1:**
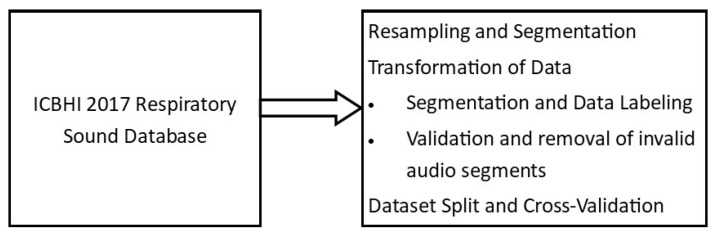
Preprocessing steps applied to the ICBHI 2017 Respiratory Sound Database.

Figure 1 shows the preprocessing steps applied to the ICBHI 2017 Respiratory Sound Database. The recorded dataset of the ICBHI 2017 Respiratory Sound Database was used in the research work, recorded at sample rates that varied from 4 KHz to 44.1 KHz. Resampling all recordings at a sampling rate of 4 KHz was deemed a safe approach because the frequency range of the signal of interest remained below 2 KHz. The resampled dataset was subsequently divided into segregated respiratory cycles and annotated. The overall duration of the respiratory cycles in a dataset ranges from 0.2 to 16 s, with a mean value of 2.7 s. The methodology reported by [15] was deployed to extract respiratory cycles of consistent duration. Specifically, cycles exceeding 2.7 s were truncated, retaining only the initial 2.7 s.

**Figure 2:**
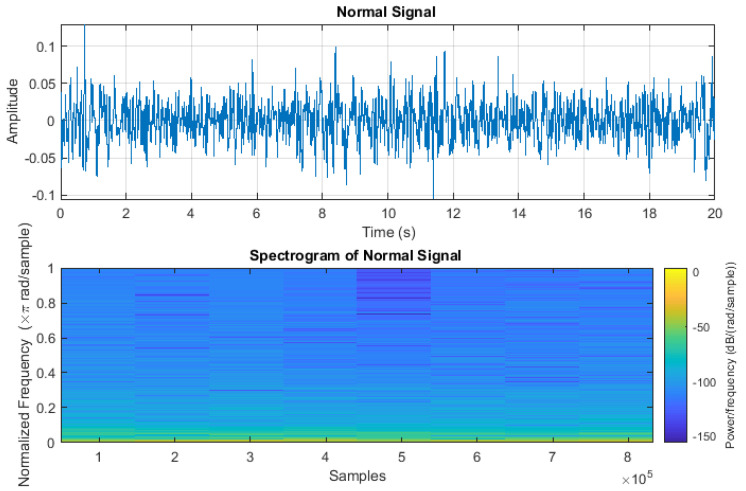
Spectrogram of lung sound that does not contain any crackles or wheezes.

In addition, the segmentation process was used to extract the necessary wheeze and crackling information from the audio signal. During the model’s training, the signals were divided into different samples to enhance their accuracy, with each sample having a duration of 20 to 25 milliseconds. Each of these samples contained either a crackle or wheeze sound. The purpose of this segmentation was to facilitate a more accurate detailed analysis and description to confirm the high accuracy of the sound signals. To further validate the quality of the signals, spectrograms of each signal were generated. Any audio segments deemed invalid—those lacking wheezing or crackling, as confirmed by the spectrogram—were discarded. This process ensures the integrity and reliability of the data. This step is shown in Figure 2. The absence of wheezing or crackling sounds in typical respiratory patterns was confirmed through waveform analysis. A continuous and periodic signal, symbolized by the wave, indicated normal respiratory cycles. The consistent recurrence of smooth waveforms served as an indicator of a normal respiratory cycle. To handle the combined crackles and wheezes category, we extracted relevant segments containing both crackles and wheezes from the audio samples. These segments were then included in the training and testing datasets alongside the other categories.

This comprehensive approach to segmentation and validation not only optimized the model performance but also ensured the integrity of the dataset for subsequent analyses and interpretations.

**Figure 3:**
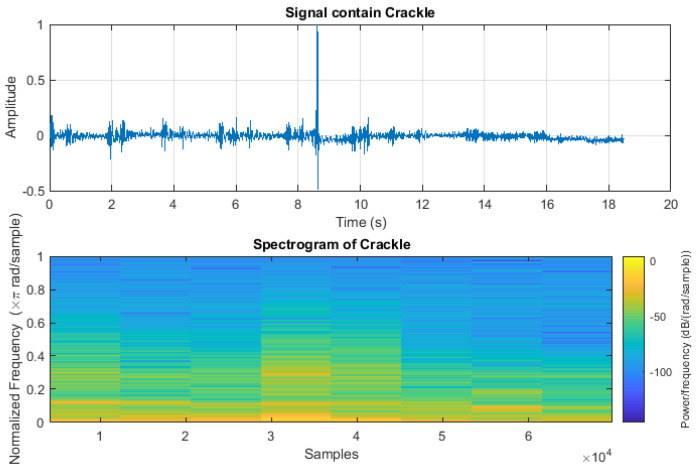
Wave plot of the audio segment containing crackle and its spectrogram.

**Figure 4:**
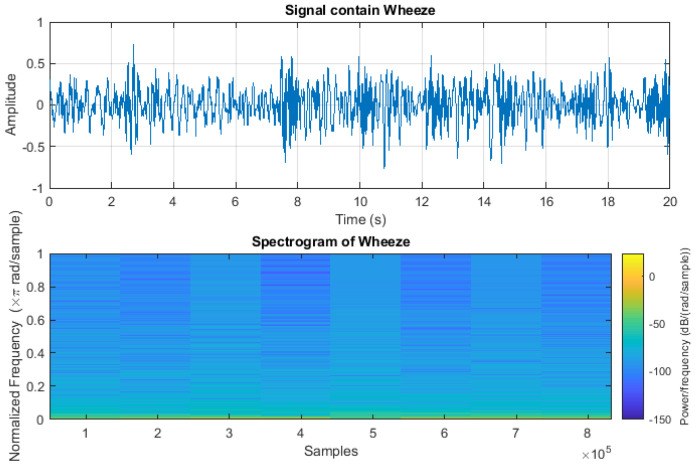
Wave plot for audio segment containing wheeze and its spectrogram.

**Figure 5:**
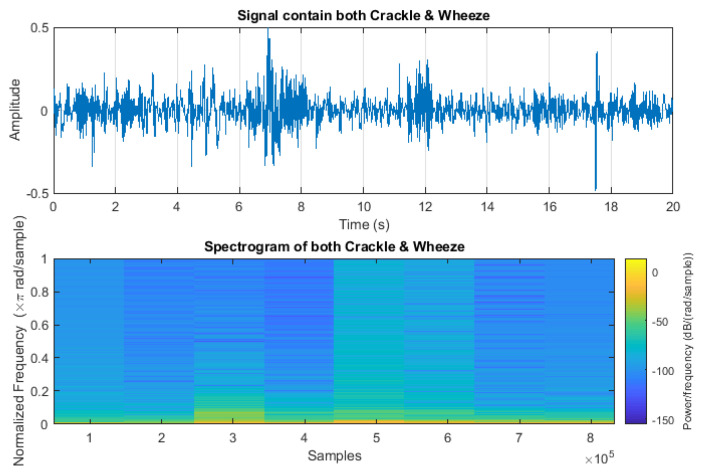
Wave plot for audio segment containing both wheeze and crackle and its spectrogram.

The crackle signal, characterized by bubbling, rattling, or clicking sounds, is shown in Figure 3. Figure 4 shows wheezes within a respiratory cycle during both inhalation and exhalation. The wave plots and spectrograms of these two categories exhibit distinct characteristics. Figure 5 shows the wave plot for an audio segment along with its spectrogram, containing both wheezes and crackles.

After segmentation and validation, the audio segments were split into two distinct sets: a training set (70%), used to train the 1D-CNN model, and a testing set (30%), reserved for evaluating its performance on unseen data. To further assess the robustness and generalization capability of our model, we utilized the k−fold cross-validation technique. This involves segmenting the training data into k subsets (folds), training the model on k−1 folds, and evaluating it on the remaining fold. The process is repeated k times, with each fold serving as the evaluation set once.

### 2.3. Convolution Neural Network (CNN)

The CNN algorithm has become a widely recognised and extensively utilized method in the domain of Deep Learning (DL). Compared to its predecessors, one notable feature of CNN is its autonomous ability to identify relevant components or features within the data without human intervention [28]. While traditional Convolutional Neural Networks (CNNs) are widely used and perform well in many deep learning tasks, their inherently two-dimensional (2D) nature can make them computationally demanding in certain applications. However, 1D-CNNs emerge as a powerful alternative, with improved computational efficiency and an enhanced performance in processing sequential data [20,23,24,25,26].

The mathematical representation of a CNN for every given signal is expressed as Equation (1).

(1) $$X\left(t\right)=\sum_{\tau =-\infty }^{\infty }x\left(\tau \right)\delta \left(t-\tau \right)$$

where δ(t) is the sequence, such that δ(0)=1 and for t≠(0), δ(t)=0.

**Figure 6:**
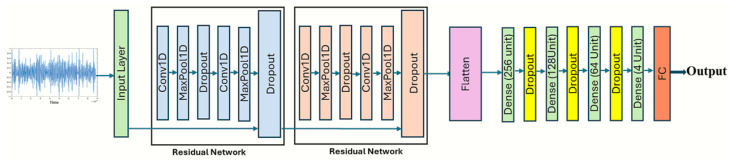
One-Dimensional Convolutional Neural Network (1D-CNN) architecture.

The generic architecture of a CNN is shown in Figure 6. The CNN’s convolutional layers are the basic structural elements responsible for extracting features from the time series or uni-dimensional signals. A series of filters, commonly known as kernels, are used to accomplish this task. These kernels, whose values are obtained through the training process, act as the impulse response of the filters [18], effectively capturing the salient characteristics of the input signal. In the proposed 1D-CNN architecture, we employ multiple convolutional layers with increasing filter sizes to capture both local and global patterns in the lung sound signals. The first convolutional layers uses a a smaller filter size to learn local features, while subsequent layers gradually increase the filter size to capture more global patterns [29]. This hierarchical approach allows for the network to learn a robust representation of the lung sounds at different scales.

To further enhance the performance of our 1D-CNN, residual connections inspired by the ResNet architecture are incorporated [9,30]. Residual connections allow the network to learn residual functions with reference to the layer inputs, enabling the training of deeper networks without degradation. By adding skip connections between convolutional layers, we facilitate the flow of information and gradients throughout the network, improving its ability to learn complex patterns in the lung sound data.

The pooling layers serve as an additional element placed directly after each convolutional layer. Pooling layers perform nonlinear down-sampling on the extracted feature maps while reducing their spatial dimension while preserving the essential information. This process helps reduce the network’s computational complexity, making it more efficient in sequential data processing. In our 1D-CNN, we employ max pooling layers to down-sample the feature maps and retain the most prominent features.

Furthermore, dropout layers are used to reduce over-fitting in the network by deactivating a subset of its neurons during training. This promotes better generalization. On the other hand, batch normalization layers are also used to enhance the performance of the training procedure by normalizing the activation of the previous layer, reducing internal covariate shift.

Finally, it is important to note that fully connected layers are a type of feedforward neural network (FNN) that is usually connected at the end of a network. These layers establish weighted connections between the overall functionality of the previous layers and the distribution of class probabilities, simplifying the network’s ability to make predictions. In our 1D-CNN, we utilize fully connected layers to map the learned features to the desired output classes (normal, crackles, wheezes, and a combination of both crackles and wheezes).

To address the class imbalance present in the lung sound dataset, the Synthetic Minority Over-sampling Technique (SMOTE) was applied to the training data [31]. SMOTE generates synthetic examples of the minority classes by interpolating between existing examples, effectively balancing the class distribution. The SMOTE algorithm can be mathematically represented as follows:

- For an individual minority class sample $x_{i}$, select one of its k-nearest neighbors $x_{j}$.
- Generate a new synthetic sample $x_{new}$ using the following equation:
  (2) $$x_{new}=x_{i}+r*\left(x_{j}-x_{i}\right)$$
  where r is a random number between 0 and 1.
- Repeat steps 1 and 2 until the desired balance between the minority and majority classes is achieved.

By applying SMOTE to the training data, we ensured that the 1D-CNN model was exposed to a balanced distribution of samples from each class, mitigating the bias towards the majority class.

To find the optimal hyperparameters for our 1D-CNN model, we used a grid search with k−fold cross-validation. Grid search is an exhaustive search technique that evaluates model performance for different combinations of hyperparameters. The hyperparameter grid is defined in Table 2.

The k−fold cross-validation technique segments the training data into k. subsets or folds, trains the model on k−1 folds, and evaluates it on the remaining fold. This process is repeated k times, with each fold serving as the validation set once. The average performance across all folds provides a robust estimate of the model’s generalization ability. For k−fold cross-validation, the training data are divided *X* into *k* disjoint subsets $X_{1}$, $X_{2}$, …, $X_{k}$ of approximately equal size. For each fold i=1,2,…,k:

(a) Train the model on $X\setminus X_{i}$ (all subsets except $X_{i}$).
(b) Evaluate the model on $X_{i}$ and compute the performance metric.

Afterward, the average performance across all *k* folds is calculated. By combining grid search with k−fold cross-validation, we can identify the optimal hyperparameters that yield the best performance on unseen data, ensuring the robustness and generalization capability of our 1D-CNN model.

The modelled 1D-CNN architecture comprises 1,610,466 parameters. The model summary of the 1D-CNN is depicted in Table 3.

The 1D-CNN model effectively categorizes the signals into the four classes (normal, crackles, wheezes, and combined crackles and wheezes) and yields a classification accuracy of 0.95. The identification of wheezing and crackles in lung sounds is typically performed by pulmonologists, who distinguish them from extraneous environmental noises such as chairs being dragged, fans in motion, or rustling paper, among others. Subsequently, a meticulously organized repository of categorized lung sound samples is generated. The classification method based on CNN is employed to discern the lung sounds and categorize them into their respective classes.

In summary, our proposed 1D-CNN architecture leverages the strengths of convolutional neural networks in capturing local and global patterns, residual connections for improved information flow, and focal loss to address class imbalance. This combination of techniques enables the network to learn robust representations of lung sounds and accurately classify them into the four categories of normal, crackles, wheezes, and combined crackles and wheezes.

In this method, the initial step involves classifying data into the following categories: normal, cracking, wheezing, and combined crackles and wheezes. This classification was performed by pulmonologists using the existing data set. Environmental artifact noises were removed from the data set due to the presence of wheezing and crackles, which were the sounds of interest. Subsequently, the model underwent training using the processed classified data set. Using a 1D-CNN demonstrated encouraging outcomes in distinguishing between crackle and wheeze respiratory sounds and discerning between crackle and wheeze sounds. The classification method based on deep learning achieved a high accuracy of 0.95 in detecting lung sounds. The categorization of abnormal sounds into distinct subtypes, such as crackles and wheezes, yielded similar results. These findings are noteworthy, particularly considering the diverse range of auditory stimuli utilized for analysis. In the context of screening and subsequent testing for respiratory infections, it is deemed satisfactory to achieve accuracies of 0.95. This research’s main contribution is utilizing a 1D-CNN prediction model to categorize respiratory sounds. The modelled 1D-CNN comprises 1,610,466 parameters resulting from the training of the 1D-CNN model. A model summary of the 1D-CNN is depicted in Table 3.

The 1D-CNN model effectively categorized the signals into the four classes—normal, wheezes, crackles, and combined crackles and wheezes—yielding a classification accuracy of 0.95. The identification of wheezing and crackles in lung sounds is typically performed by pulmonologists, who distinguish them from extraneous environmental noises such as chairs being dragged, fans in motion, or rustling paper, among others. Subsequently, a meticulously organized repository of categorized lung sound samples was generated. The classification method based on CNN was employed to discern and categorize the lung sounds into the respective classes.

In summary, our proposed 1D-CNN architecture leverages the strengths of convolutional neural networks in capturing local and global patterns, residual connections for improved information flow, and focal loss to address class imbalance. This combination of techniques enables the network to learn robust representations of lung sounds and accurately classify them into the four categories of normal, wheezes, crackles, and combined crackles and wheezes.

## 3. Results and Discussion

The 1D-CNN model proposed in this study was trained on the extended dataset, including all four categories of lung sounds. The model performance was assessed using accuracy, precision, recall, and F1-score metrics for each class. Table 4 displays the classification outcomes for the four categories.

The proposed model achieved an overall accuracy of 0.95, indicating its effectiveness in categorizing lung sounds across the four classes. The normal and crackles categories attained the highest F1 scores of 0.97 and 0.95, respectively. The crackles and wheezes categories also showed a strong performance, achieving F1 scores of 0.95 and 0.94 respectively. However, the combined category of crackles and wheezes posed a greater challenge, resulting in a slightly lower F1 score of 0.89. This can be attributed to the complexity of accurately identifying both crackles and wheezes within the same audio segment.

**Figure 7:**
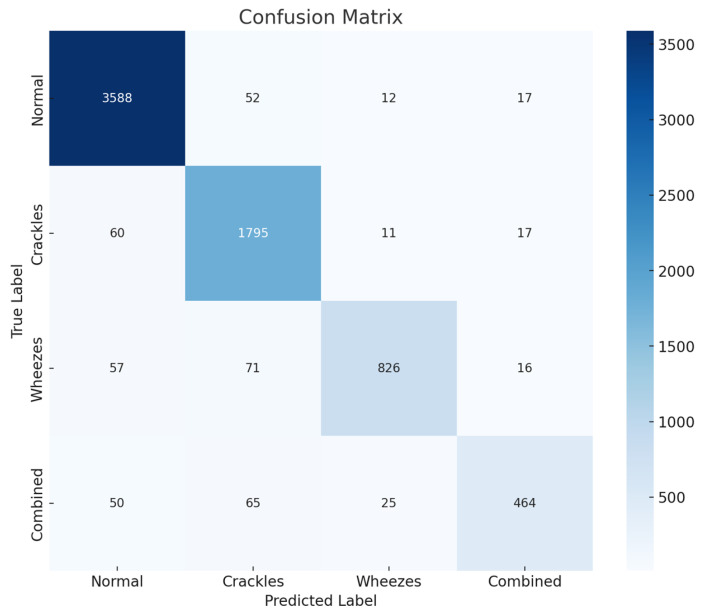
Confusion matrix for the four-class classification task.

Figure 7 presents the confusion matrix for the four-class classification task. The matrix provides insight into the model performance, highlighting correct classifications along the diagonal and misclassifications in off-diagonal entries. The normal category exhibits the highest number of corrected classifications (3588), followed by crackles (1795), wheezes (826), and combined (464). The confusion matrix also shows misclassifications, especially in the crackles, wheezes, and combined categories. This indicates the difficulty in distinguishing between these overlapping sounds.

**Figure 8:**
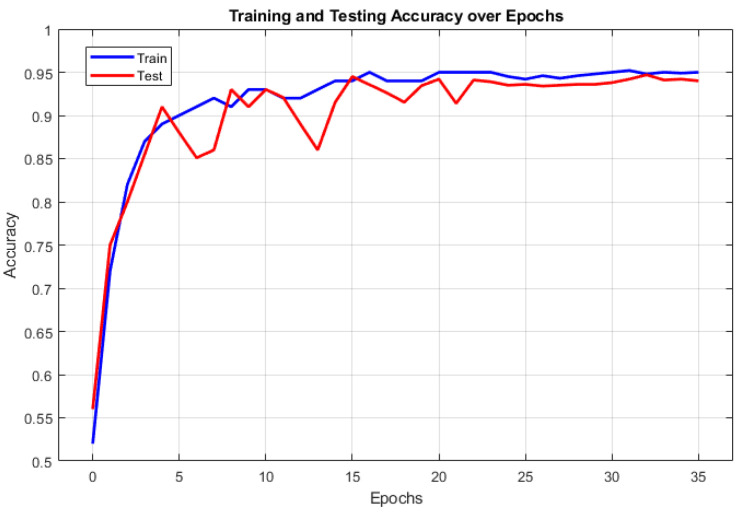
Training and testing accuracy curves.

**Figure 9:**
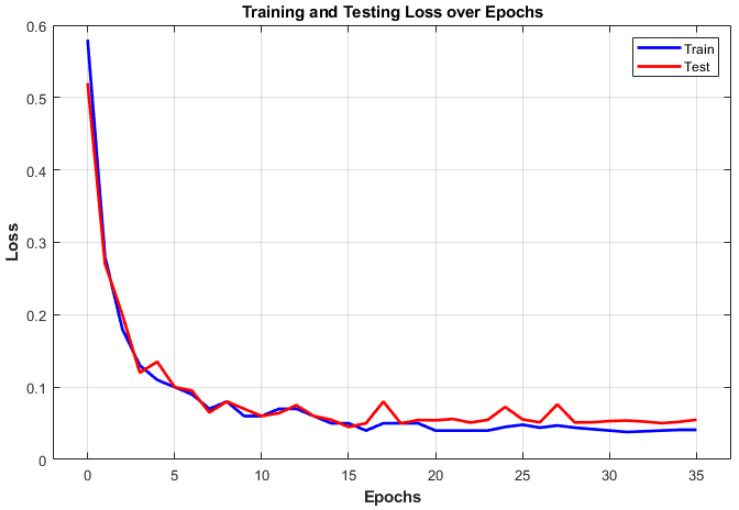
Training and testing loss curves.

The training and testing accuracy curves for the 1D-CNN model are depicted in Figure 8. The blue line represents the training accuracy, which steadily increases as the model learns from the data. The red line represents the testing accuracy, which closely tracks the training accuracy, showing minimal overfitting. The model achieves high accuracy on both training and testing datasets, showing its ability to generalize effectively to new data. The training and testing loss curves of the 1D-CNN model are shown in Figure 9. The training loss is represented by the blue line, whereas the red line corresponds to the testing loss. Both curves exhibit a decreasing trend, suggesting the model effectively learns from the data and reduces classification errors. The convergence of these curves further validates the model’s robustness and ability to avoid overfitting.

### 3.1. Comparison with the State-of-the-Art Research

Table 5 shows the performance of the proposed 1D-CNN model in other studies that have classified all four categories of lung sounds from the ICBHI 2017 dataset. The proposed 1D-CNN model outperforms the state-of-the-art methods in classifying all four categories of lung sounds, achieving an accuracy of 0.95. Pham et al.’s [16] study using the CNN-MoE framework and the work by Nguyen and Pernkopf [15] using co-tuning and stochastic normalization techniques achieved accuracies of 0.93 and 0.94, respectively. The studies conducted by the Demir et al. [14] and Cinyol et al. [32] used the CNN model for lung sound detection and obtained accuracies of 0.92 and 0.94, respectively. The lightweight CNN model proposed by Wanasinghe et al. [10] also shows a comparable performance, with an accuracy of 0.94.

The strengths of the proposed 1D-CNN approach lie in its ability to effectively capture temporal patterns and distinguish between different lung sound categories. The 1D convolutions enable the model to learn discriminative features directly from the raw audio signals, reducing the need for manual feature engineering. However, the limitations of the study include the challenges in accurately classifying the combined crackles and wheezes category, which exhibits some overlap with the individual crackles and wheezes categories.

### 3.2. K−Fold Cross-Validation

To further evaluate the robustness and generalization capability of the 1D-CNN model, a k−fold cross-validation was performed. Table 6 presents the results of the 5−fold and 10−fold cross-validation experiments.

The results of the cross-validation experiments demonstrate the model’s robustness and ability to generalize well across different data partitions. The 5−fold cross-validation achieved an accuracy of 0.9710, while the 10−fold cross-validation achieved an accuracy of 0.9730. The sensitivity, specificity, precision, and F1 scores also remained consistently high, further validating the model’s performance.

The proposed research represents an important contribution to the field of respiratory sound analysis, as it successfully applies 1D-CNN for the classification of lung sounds into four clinically relevant categories. The achieved accuracy of 0.95 shows promising results for the automatic diagnosis of respiratory diseases. The proposed approach has the potential to increase the accuracy and efficiency of respiratory disease diagnosis by accurately distinguishing between normal sounds, crackles, wheezes, and their combination.

From a therapeutic perspective, the accurate categorization of respiratory sounds has the potential to identify respiratory diseases, allowing for prompt treatment and better patient care. The proposed research highlights the potential for the automated analysis of respiratory sounds, which could be an important asset in healthcare, assisting medical professionals in making informed decisions and improving patient outcomes.

## 4. Conclusions and Future Work

This study focused on designing a Clinical Decision Support System (CDSS) for the early detection of respiratory disorders by analyzing lung sounds using a one-dimensional convolutional neural network (1D-CNN) model. The proposed system demonstrated an accurate and reliable classification of lung sounds into four categories: normal, crackles, wheezes, and combined crackles and wheezes. The 1D-CNN model achieved an overall accuracy of 0.95, outperforming state-of-the-art methods.

The main contributions of this study is in the effective use of 1D-CNN for classifying lung sounds into clinically relevant categories, a comprehensive evaluation of the model performance using different metrics, and a comparison with existing studies in the field. The proposed approach has the potential to significantly enhance the accuracy and efficiency of diagnosing respiratory diseases by accurately identifying normal and abnormal lung sounds, as well as differentiating between specific types of abnormalities (crackles, wheezes, and their combination).

The application of deep learning algorithms, particularly 1D-CNN, in analyzing lung sounds has yielded promising results for the automated diagnosis of respiratory diseases. The high accuracy achieved by the proposed system, as evidenced by the results and cross-validation experiments, underscores its potential for real-world clinical applications.

To address the issue of class imbalances in the lung sound dataset, the Synthetic Minority Over-sampling Technique (SMOTE) is used on the training data. This technique balanced the class distribution, enabling the 1D-CNN model to learn from a more representative set of samples. Furthermore, a grid search with k−fold cross-validation was utilized to identify the optimal hyperparameters for the model, ensuring its robustness and generalization capability, as demonstrated by the high accuracy of 0.95 on the test set.

In future work, we plan to conduct comprehensive ablation studies to quantify the impact of residual connections and other key components of our model. We believe this will provide valuable insights into the contribution of each architectural element to the overall performance of our 1D-CNN model in lung sound classification. Additionally, we are planning to explore the integration of advanced data augmentation techniques, such as generative adversarial networks (GANs), to further enhance the diversity and quality of the training data.

Future studies should also focus on validating the performance of the proposed model using larger and more diverse datasets, taking into account factors such as patient demographics, recording equipment, and environmental noise. Additionally, we are planning to integrate the proposed CDSS into existing clinical workflows through prospective studies and clinical trials. This will help us assess its impact on patient outcomes in real-world healthcare settings.

In conclusion, this study presented a novel approach to respiratory disease diagnosis using a 1D-CNN-based model for lung sound classification. The proposed system demonstrates high accuracy in categorizing lung sounds into normal, crackles, wheezes, and combined crackles and wheezes, outperforming state-of-the-art methods. The integration of this technology into clinical practice has the potential to revolutionize the diagnosis and management of respiratory disorders, ultimately improving patient outcomes and enhancing the efficiency of healthcare systems. However, further research and validation in real-world settings, as well as the development of user-friendly interfaces and guidelines, are necessary to fully realize the potential of this approach. As the field of digital health continues to evolve, the integration of advanced machine learning techniques, such as the proposed 1D-CNN model, into clinical decision support systems holds great promise for transforming respiratory care and advancing the early detection and treatment of respiratory diseases. 

## Figures and Tables

**Table 1:** Details of the distribution of the lung sounds in the ICBHI 2017 Database [27].

Participants	128
Recordings	920
Normal	3642
Crackle	1864
Wheeze	886
Combined (crackles and wheezes)	506
Respiratory cycles	6898

**Table 2:** Hyperparameter grid for the 1D-CNN model.

Hyperparameter		Values	
Number of Filters:	32	64	128
Kernel Size:	3	5	7
Dropout Rate:	0.2	0.3	0.4
Num units:	64	128	256
Learning Rate:	0.0001	0.001	0.01

**Table 3:** The model summary of the 1D-CNN.

Layer (Type)	Output Shape	Param#
input_1(Input layer)	(None, 8000, 1)	0
conv1d (CONV1D)	(None, 7988, 8)	112
max_pooling1d {MaxPoolling1D}	(None, 2662, 8)	0
dropout (Dropout)	(None, 2662, 8)	0
conv1d_(CONV1D)	(None, 2652, 16)	1424
max_pooling1d_1 (MaxPooling1D)	(None, 884, 16)	0
dropout_1 (Dropout)	(None, 884, 16)	0
conv1d_2 (Conv1D)	(None, 876, 32)	4640
max_pooling1d_2 (MaxPooling1D)	(None, 292, 32)	0
dropout_2 (Dropout)	(None, 292, 32)	0
conv1d_3 (Conv1D)	(None, 286, 64)	14,400
max_pooling1d_3 {MaxPooling1D}	(None, 95, 64)	0
dropout_3 (Dropout)	(None, 95, 64)	0
flatten (Flatten)	(None, 6080)	0
dense (Dense)	(None, 256)	1,556,736
dropout_4 (Dropout)	(None, 256)	0
dense_1 (Dense)	(None, 128)	32,896
dropout_5 (Dropout)	(None, 128)	0
dense_2 (Dense)	(None, 64)	8256
dropout_6 (Dropout)	(None, 64)	0
dense_3 (Dense)	(None, 4)	260

**Table 4:** Classification Results for Four Lung Sound Categories.

Category	Accuracy	Precision	Recall	F1 Score
Normal	0.97	0.97	0.98	0.97
Crackles	0.95	0.92	0.95	0.95
Wheezes	0.95	0.96	0.91	0.94
Combined	0.93	0.90	0.89	0.89

**Table 5:** Comparison with state-of-the-art research (four-class classification).

Study	Method	Accuracy
Proposed study	1D-CNN	0.95
Pham et al. [16]	CNN-MoE	0.93
Demir et al. [14]	CNN	0.92
Cinyol et al. [32]	CNN	0.94
Nguyen and Pernkopf [15]	Co-tuning + SN	0.94
Petmezas et al. [9]	CNN − LSTM	0.93
Acharya and Basu [11]	CNN + LSTM	0.94
Wanasinghe et al. [10]	Lightweight CNN	0.94

**Table 6:** K−fold cross-validation results.

Metric	5−Fold Cross-Validation	10−Fold Cross-Validation
Accuracy	0.9710	0.9730
Sensitivity	0.9820	0.9840
Specificity	0.9570	0.9600
Precision	0.9570	0.9600
F1 Score	0.9820	0.9840

## Data Availability

The initial data set that was used is available on [27]. The modified data used for the design of CDSS can be requested from the corresponding authors.

## Abbreviations

The following abbreviations are used in this manuscript:

CNN	Convolutional Neural Network
1D-CNN	One-Dimensional Convolutional Neural Network
CDSS	Clinical Decision Support System
LD	Linear Dichroism
